# Interleukin-6 displays lung anti-inflammatory properties and exerts protective hemodynamic effects in a double-hit murine acute lung injury

**DOI:** 10.1186/s12931-017-0553-6

**Published:** 2017-04-19

**Authors:** Guillaume Voiriot, Keyvan Razazi, Valérie Amsellem, Jeanne Tran Van Nhieu, Shariq Abid, Serge Adnot, Armand Mekontso Dessap, Bernard Maitre

**Affiliations:** 1 INSERM, Unité U955 (Institut Mondor de Recherche Biomédicale), Créteil, France; 2 Faculté de Médecine, Groupe de recherche clinique CARMAS, Université Paris Est, Créteil, France; 3 Groupe Henri Mondor-Albert Chenevier, Hôpital Henri Mondor, Service de Réanimation Médicale, AP-HP, Créteil, France; 4 Groupe Henri Mondor-Albert Chenevier, Hôpital Henri Mondor, Service d’Anatomie et Cytologie Pathologiques, AP-HP, Créteil, France; 5 Groupe Henri Mondor-Albert Chenevier, Hôpital Henri Mondor, Service des Explorations Fonctionnelles, AP-HP, Créteil, France

**Keywords:** Interleukin-6, Acute lung injury, Acute respiratory distress syndrome, Mechanical ventilation, Pulmonary hypertension, Nitric oxide synthase

## Abstract

**Background:**

Interleukin 6 (IL-6) is a predictive factor of poor prognosis in patients with acute respiratory distress syndrome (ARDS). However, its acute pulmonary hemodynamic effects and role in lung injury have not been investigated in a clinically relevant murine model of ARDS.

**Methods:**

We used adult C57Bl6 wild-type (WT) and IL-6 knock-out (IL-6KO) mice. The animals received intravenous recombinant human IL-6 (rHuIL-6) or vehicle followed by intratracheal lipopolysaccharide (LPS) or saline before undergoing low tidal volume mechanical ventilation (MV) for 5 h. Before sacrifice, right ventricular systolic pressure and cardiac output were measured and total pulmonary resistance was calculated. After sacrifice, lung inflammation, edema and injury were assessed with bronchoalveolar lavage (BAL) and histology. In other experiments, right ventricular systolic pressure was recorded during hypoxic challenges in uninjured WT mice pretreated with rHuIL-6 or rHuIL-6 + non-selective nitric oxide synthase inhibitor L-NAME or vehicle.

**Results:**

IL-6KO_(LPS+MV)_ mice showed a faster deterioration of lung elastic properties and more severe bronchoalveolar cellular inflammation as compared to WT_(LPS+MV)_. Treatment with rHuIL-6 partially prevented this lung deterioration. Total pulmonary resistance was higher in IL-6KO_(LPS+MV)_ mice and this increase was abolished in rHuIL-6-treated IL-6KO mice. Finally, rHuIL-6 reduced hypoxic pulmonary vasoconstriction in uninjured WT mice, an effect that was abolished by co-treatment with L-NAME.

**Conclusions:**

In a double-hit murine model of ARDS, IL-6 deficient mice experienced more severe bronchoalveolar cellular inflammation as compared to wild-type littermates. Furthermore, IL-6 deficiency caused marked acute pulmonary hypertension, which may be, at least partially, due to vasoactive mechanisms. A dysregulation of nitric oxide synthase may account for this observation, a hypothesis that will need to be investigated in future studies.

## Background

Acute Respiratory Distress Syndrome (ARDS) is a life-threatening syndrome consisting of acute onset respiratory failure caused by noncardiogenic edema and characterized by hypoxemia and radiological bilateral lung infiltrates [1]. The pathology includes alveolocapillary membrane abnormalities due to various agents either inhaled or delivered to the lungs via the blood circulation [2]. The pathogenesis involves a potent inflammatory response, involving a complex group of mediators including interleukin-6 (IL-6). This pleiotropic cytokine is produced at sites of tissue inflammation and released into the circulation by a variety of different cell types, including macrophages, lymphocytes, endothelial cells, epithelial cells and fibroblasts [3] during sepsis and acute organ injuries. IL-6 acts as a major pro-inflammatory mediator for the induction of the acute phase response [4], leading to a wide range of local and systemic changes including fever, leucocytes recruitment and activation, hepatic regeneration and hemodynamic effects. Considering the key role of IL-6 in mediating the acute phase response, its value as a prognostic biomarker in sepsis and various acute organ injuries has been extensively investigated in clinical and experimental studies. Plasma and/or broncho-alveolar levels of IL-6 have been identified as early biomarkers of lung injury [5–8] and predictive factors of prolonged mechanical ventilation, organ dysfunctions, morbidity and mortality in ARDS [9–12]. However, whether the increased plasma or tissue levels of IL-6 observed in sepsis and ALI/ARDS, contribute to organ injury, prevent it or merely describe it, remains controversial [13]. Indeed, beneficial anti-inflammatory effects of IL-6 have been shown in experimental lung injury [14–16]. This hypothesis has not been tested in a lung injury model closely relevant to the clinical situation. Furthermore, the pulmonary hemodynamic effects of IL-6 in ARDS remain unclear, even though pulmonary vascular dysfunction is a major hallmark of ARDS [17]. In this study, we used a double-hit, clinically relevant murine model of ARDS combining lipopolysaccharide (LPS) aspiration followed by 5-h low tidal volume mechanical ventilation. We applied this model to IL-6 gene knock-out mice to assess the effects of IL-6 during ARDS on respiratory mechanics, lung inflammation, and right-sided ventricular hemodynamics.

## Methods

### Animals

Mice lacking IL-6 (IL-6^−/−^) were generated by homologous recombination on the C57Bl/6 and IL-6^−/−^ genetic background [18]. The wild-type IL-6^+/+^ mice (WT mice) and mutant homozygous knock-out IL-6^−/−^ mice (IL-6KO mice) were adult male littermates obtained by breeding heterozygous mutants. All animal care and procedures were performed after approval of the Institutional Animal Care Committee, in accordance with official guidelines of the French Ministry of Agriculture for the experimental use of animals.

### Animal protocol

Mice were anesthetized with inhaled 5% isoflurane (Abbott, Rungis France) and were maintained spontaneously breathing. They received retro-orbital intravenous injections [19] of either 3 μg/kg of recombinant human IL-6 (rHuIL-6, PromoCell, Heidelberg, Germany) or vehicle (saline). Following the injection, 2 μl/g of body weight volume instillate consisting of 2 μg/μl of Escherichia coli 055:B5-derived LPS (Sigma-Aldrich Chimie, Lyon, France) or vehicle (saline) was administered into the distal part of the oropharynx and aspirated into the lower respiratory tract. The selection of rHuIL-6 doses was based on previously published studies with intravenous rHuIL-6 in mouse models of sepsis [20] and acute myocardial infarction [21]. Following recovery from anesthesia, the mice were returned to their cage with free access to water and food.

Eighteen hours later, mice were anesthetized with a combination of intraperitoneal pentobarbital (30 μg/g of body weight, Hospira, Meudon La Forêt, France) and inhaled 5% isoflurane (Abbott, Rungis France). Following a second intravenous injection of rHu IL-6 (identical to the first injection) or vehicle, the larynx was surgically exposed and the trachea intubated orally under direct vision with a metal cannula (internal diameter of 1 mm, Harvard Apparatus, Les Ulis, France). The tracheal cannula was properly secured with surgical thread (Ethicon 3–0, Ethicon, Auneau, France) before being connected to a mechanical ventilator. The cervicotomy was closed with surgical thread (Ethicon 6.0, Ethicon, Auneau, France).

### Mechanical ventilation

The ventilator regimen has been extensively detailed elsewhere [22]. Briefly, mice were ventilated in the supine position using humidified gas (20 mgH2O/L absolute humidity, MR410 humidifier, Fischer & Paykel Healthcare, Courtaboeuf, France), with a tidal volume of 8 mL/kg of body weight, a respiratory rate of 180/min, 3 cmH2O end-expiratory pressure, and FiO2 of 0.5, by means of a computer-driven small-animal ventilator (flexiVent, Scireq, Montreal, Canada). Mechanical ventilation lasted 5 h with continuous anesthesia maintained by 1.5% isoflurane and muscle paralysis using intraperitoneal pancuronium given at the onset of mechanical ventilation and then every two hours (0.8 μg/g of body weight, Organon, Puteaux, France) to ensure passive mechanical conditions. Mice received intraperitoneal warm fluid boluses (5% dextrose with 9 g/L NaCl) at the onset (20 μL/g of body weight) and every hour (10 μL/g of body weight) during mechanical ventilation.

### Experimental design

The experimental design included six groups: WT_(MV)_ (WT receiving saline instillation and vehicle injections and undergoing mechanical ventilation (MV)), WT_(LPS+MV)_ (WT mice receiving LPS instillation and vehicle injections and undergoing mechanical ventilation), WT + rHuIL-6_(LPS+MV)_ (WT mice receiving LPS instillation and rHuIL-6 injections and undergoing mechanical ventilation), IL-6KO_(MV)_ (IL-6KO mice receiving saline instillation and vehicle injections and undergoing mechanical ventilation), IL-6KO_(LPS+MV)_ (IL-6KO mice receiving LPS instillation and vehicle injections and undergoing mechanical ventilation) and IL-6KO + rHuIL-6_(LPS+MV)_ (IL-6KO mice receiving LPS instillation and rHuIL-6 injections undergoing mechanical ventilation (MV).

### Model assessment

To contrast the effects of the double-hit challenge (LPS + mechanical ventilation) with those of either LPS administration alone, we subjected wild-type C57BL6 adult male mice to LPS or vehicle (saline) aspiration without mechanical ventilation. We then assessed lung injury and measured total cell count, and total protein, IL-6, TNFα and MIP-2 concentrations in BAL fluid as described below.

### Respiratory mechanics

Special features of the flexiVent ventilator include a continuous monitoring of airway pressures and a precision computer-controlled piston capable of accurately measuring the delivered volume (with appropriate corrections for gas compression) and producing any desired waveform, allowing respiratory mechanics assessment with the forced oscillation technique (FOT) and pressure-volume curves [22]. Following a stabilization period of 5 min, mice were inflated twice to a transrespiratory pressure of 30 cmH_2_O to establish a standard volume history. Peak inspiratory pressure was measured at initiation of mechanical ventilation, before and after volume history standardization, and then hourly. Respiratory system dynamic compliance and elastance were measured using the single frequency FOT at initiation of mechanical ventilation, before and after volume history standardization, and then hourly to capture the time course and detailed response to mechanical ventilation. The respiratory system quasi-static compliance was measured using a pressure-driven pressure-volume curve at start (before and after volume history standardization) and end of mechanical ventilation. Before the end of the timed ventilator protocol, mice underwent hemodynamic measurements [22].

### Hemodynamic measurements

Both the right jugular vein and left carotid artery were isolated through the cervical midline incision using a stereomicroscope (Leica MZ 7.5, Leica Microsystems, Nanterre, France). An ultra-miniature 0.47 mm high fidelity pressure transducer catheter (SPR-671, Millar Instruments, Houston, TX) was inserted into the right jugular vein and advanced into the right ventricle. The micromanometer was calibrated in vitro, firstly electronically and secondly against a column of mercury with the reference zero level taken at mid chest. Right ventricular systolic pressure was measured during a short end-expiratory ventilatory pause using a Gould transducer (Gould, Cleveland, USA) and a Notocord system (Emka Technologies, Paris, France). Cardiac output was measured with the transpulmonary thermodilution technique [23]. Briefly, a 0.34 mm external diameter thermistor microprobe (Columbus Instruments, Columbus, OH) was inserted into the left carotid artery and advanced into the aortic arch, where changes in aortic blood temperature were measured. A 0.5mm external diameter catheter placed in the right jugular vein was advanced into the right atrium for bolus injection of 20 μL of NaCl 9 g/L solution at 20°C. Five consecutive cardiac output measurements were obtained using the Cardiomax-III system (Columbus Instruments) and total pulmonary resistance was calculated as the ratio of right ventricular systolic pressure to cardiac output.

### Specimen collection

At the end of the timed ventilation protocol and after completion of hemodynamic measurements, mice were exsanguinated via sectioning of the left carotid artery. Arterial blood gases were determined in five WT_(LPS+MV)_ group mice. Bronchoalveolar lavage (BAL) was performed with four separate 0.5 mL aliquots of saline at 20 °C followed by lung fixation (paraformaldehyde 4%, inflation pressure 25 cmH_2_O for 1 min) and paraffin embedding. The total cell count of BAL fluid was determined from a fresh BAL fluid specimen using a Malassez hemocytometer. The BAL fluid was centrifuged (1600 rpm, 7 min at 4°C), the cell pellet was diluted in saline, and differential cell counts were done in four WT_(LPS+MV)_ mice and four IL-6KO_(LPS+MV)_ mice on cytocentrifuge preparations (Cytospin 3; Shandon Scientific, Cheshire, UK) stained with Diff-Quick (Baxter Diagnostics, McGaw Park, IL). Cell-free supernatants were stored at −80°C for subsequent duplicate measurements of total protein (Bio-Rad Protein Assay) and cytokine concentrations [macrophage inflammatory protein 2 (MIP-2), tumor necrosis factor α (TNF-α) and IL-6 using an ELISA immunoassay kit, (R&D systems, Abingdon, UK) performed in accordance with manufacturer’s instructions.

### Histological analysis

Paraffin lungs blocks were sectioned at 5μm thickness and mounted on glass slides. After staining with hematoxylin and eosin, all slides were examined under a light microscope by an experienced pathologist in a blinded fashion. Lung injury was assessed using a histological scoring system designed to evaluate the heterogeneous lesions of lung injury and adapted from previously published scores [24, 25]. Three pathological processes (edema, congestion/hemorrhage and leukocyte infiltration) were scored in the two main non-alveolar structures of the lung (veins and bronchi/arteries) using the two following scales: 1) an intensity scale (0 to 4: 0 if absent, 1 if mild, 2 if moderate, 3 if severe, and 4 if very severe), and 2) an extension scale (0 if absent, 1 if <25% of lung involvement, 2 if 25–50% of lung involvement, 3 if 50–75% of lung involvement, and 4 if >75% of lung involvement). Three pathological processes (thickness of the alveolar wall, neutrophil recruitment in airspace, and macrophage aggregation in airspaces) were scored in the alveoli using the same scales as above. A composite score of intensity and extension (intensity score times extension score) was calculated for each pathological process in each structure. The resulting values (0 to 16) were added to yield partial scores of edema in veins and bronchi/arteries (0 to 32), congestion/hemorrhage in veins and bronchi/arteries (0 to 32), leukocyte infiltration (0 to 48, associating leukocyte infiltration in veins and bronchi/arteries and thickness of the alveolar wall) and alveolitis (0 to 32, associating neutrophil aggregation and macrophage aggregation in airspaces). Finally, the four partial scores were added to yield a global lung injury score (0 to 144).

### Acute hypoxic challenge

The effects of IL-6 on pulmonary vascular reactivity were evaluated using acute hypoxic challenge. Briefly, three groups of wild-type adult male C57Bl6 mice were randomly assigned to receive rHuIL-6 + L-NAME (Nω-nitro-L-arginine methyl ester hydrochloride), rHuIL-6 + vehicle or vehicle + vehicle administered as follows: rHuIL-6 (3μg/kg of body weight) or vehicle (NaCl 9 g/L) via retro-orbital intravenous injection and L-NAME (50 μg/g of body weight, Sigma-Aldrich Chimie, Lyon, France) or vehicle (NaCl 9 g/L) intraperitoneally. Five hours later, mice were anesthetized and intubated for mechanical ventilation. A Millar transducer catheter was immediately inserted and advanced into the right ventricle. After thirty minutes of stabilization, the right ventricular systolic pressure was continuously recorded during four consecutive hypoxic challenges (FiO2 of 0.08 during 2 min, followed by 5 min of reoxygenation).

### Statistics

The data were analyzed using the SPSS Base 18.0 statistical software package (SPSS Inc, Chicago, IL). Normality of continuous data was assessed with the Kolmogorov Smirnov test. Because not all data sets were normally distributed we used the median [1st quartile - 3rd quartile] for descriptive statistics unless otherwise stated. Independent samples were compared using the Kruskal-Wallis test followed by a pairwise Mann-Whitney test, with correction for multiple testing by the Benjamini-Hochberg false discovery rate test. Two-tailed *p* values smaller than 0.05 were considered significant.

## Results

### Animals and procedures

Mice were 18 [14–22] weeks old and weighted 26 [25–29] g. Each group included 8 to 10 animals. All animals survived the protocol.

### Model assessment

**Fig. 1:**
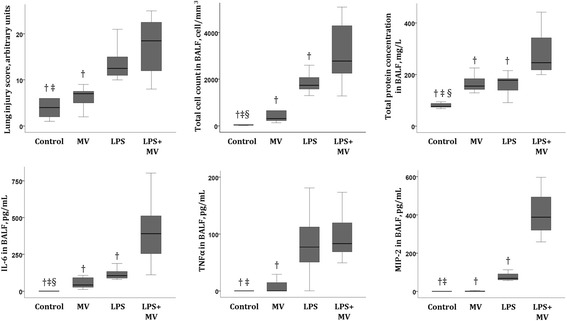
Bronchoalveolar lavage data and lung histological analysis in wild-type adult mice subjected to lipopolysaccharide (LPS) aspiration, followed by 5-h low tidal volume mechanical ventilation (MV) and their control groups (four groups: control, MV, LPS and LPS + MV; five comparisons: control *vs.* MV, control *vs.* LPS, control *vs.* LPS + MV, MV *vs.* LPS + MV, LPS *vs.* LPS + MV). The lung injury score, total cell count, and total protein, IL-6, TNFα and MIP-2 concentrations in BAL fluid were assessed. The symbols §, ‡ and † denote Benjamini-Hochberg corrected p values <0.05 of Mann-Whitney pairwise comparisons (after Kruskal Wallis test) between the group so marked and the group that received MV alone, LPS alone and the combination LPS + MV, respectively. Data are presented as box and whiskers plots. *N* = 8–10 animals per group

Results are reported in Fig. 1. As compared to mice undergoing mechanical ventilation alone (MV group), mice subjected to both LPS and mechanical ventilation (LPS + MV group) showed higher lung injury score, higher total cell count and higher total protein, IL-6, TNFα and MIP-2 concentrations in BAL fluid. As compared to mice receiving LPS alone (LPS group), mice subjected to both LPS and mechanical ventilation (LPS + MV group) showed a higher total cell count and higher total protein, IL-6, and MIP-2 concentrations in BAL fluid.

### Respiratory mechanics

**Fig. 2:**
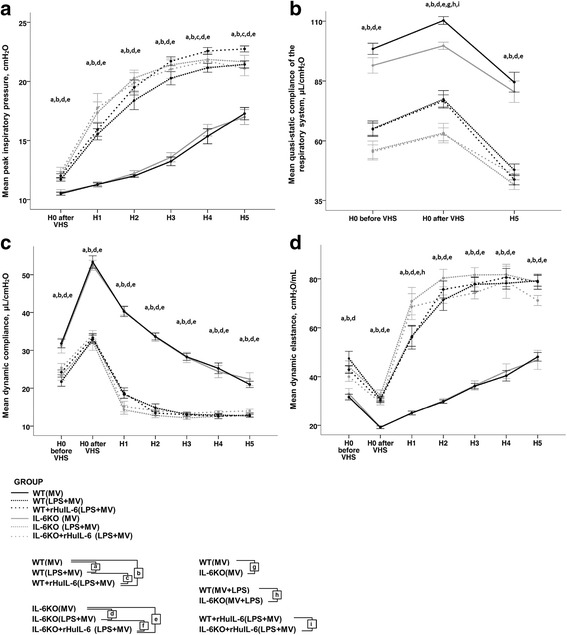
Respiratory mechanics during 5-h (H0 to H5) low tidal volume mechanical ventilation (MV) following either lipopolysaccharide (LPS) or vehicle (saline) aspiration in wild type (WT) and interleukin-6 knock-out (IL-6KO) mice receiving either recombinant human interleukin-6 (rHuIL-6) or vehicle (saline). The following respiratory system properties are shown **A.** Peak inspiratory pressures. **B.** Quasi-static compliance of the respiratory system calculated using a pressure-volume curve. **C.** Dynamic compliance of the respiratory system calculated using the single frequency forced oscillation technique. **D.** Dynamic elastance of the respiratory system calculated using the single frequency forced oscillation technique. Volume history standardization (VHS) consisted in two inflations to an airway pressure of 30 cmH2O. The letters a, b, c, d, e, f, g, h and i denote Benjamini-Hochberg corrected p values <0.05 of Mann-Whitney pairwise comparisons (following Kruskal Wallis test): WT_(MV)_ *vs.* WT_(LPS+MV)_, WT_(MV)_ *vs.* WT + rHuIL-6_(LPS+MV)_, WT_(LPS+MV)_ *vs.* WT + rHuIL-6_(LPS+MV)_, IL-6KO_(MV)_ *vs.* IL-6KO_(LPS+MV)_, IL-6KO_(MV)_ *vs.* IL-6KO + rHuIL-6_(LPS+MV)_, IL-6KO_(LPS+MV)_ *vs.* IL-6KO + rHuIL-6_(LPS+MV)_, WT_(MV)_ *vs.* IL-6KO_(MV)_, WT_(LPS+MV)_ *vs.* IL-6KO_(LPS+MV)_ and WT + rHuIL-6_(LPS+MV)_ *vs.* IL-6KOrHuIL-6_(LPS+MV)_ respectively. Data are presented as mean and standard error of the mean. *N* = 8 to 10 animals per group

The ventilator settings allowed a tight control of minute ventilation, and partial arterial CO_2_ pressure as determined in five animals in the WT_(LPS+MV)_ group (39 [33–43] mmHg). Results of airway pressure monitoring, forced oscillation techniques and pressure-volume curves are shown in Table 1 and Fig. 2. Baseline respiratory mechanics differed slightly between WT and IL-6KO mice. We found a lower quasi-static compliance in IL-6KO mice as compared to WT mice (101 [97–103] *vs.* 108 [106–112]; *p* < 0.001) at initiation of mechanical ventilation (after volume history standardization) (Fig. 2b), whereas we noted no differences in other parameters of respiratory mechanics. This phenotypic difference in quasi-static compliance between WT and IL-6KO mice was also observed in mice subjected to LPS aspiration with or without injections of rHuIL-6.

**Table 1:** Respiratory mechanics at start (H0) and after a 5-h (H5) of low tidal volume mechanical ventilation (MV) following a lipopolysaccharide (LPS) or vehicle (saline) aspiration in wild type (WT) and interleukin-6 knock-out (IL-6KO) mice, receiving either recombinant human interleukin-6 (rHuIL-6) or vehicle (saline)

	WT_(MV)_	WT_(LPS+MV)_	WT + rHuIL-6_(LPS+MV)_	IL-6KO_(MV)_	IL-6KO_(LPS+MV)_	IL-6KO + rHuIL-6_(LPS+MV)_
Peak Pr H0 post-VHS	10.5 [10.3–11.0]	11.6 [11.3–12.0]	12.0 [11.6–12.5]	10.7 [10.1–10.9]	11.6 [11.3–12.4]	12.0 [11.4–13.6]
Peak Pr H5	17.7 [16.3–18.3]	21.5 [20.5–22.0]	22.6 [22.3–23.5]	17.3 [15.6–18.5]	21.3 [20.0–22.5]	20.9 [20.6–22.4]
Cstat H0 pre-VHS	96 [93–106]	67 [54–73]	65 [58–69]	92 [82–99]	51 [47–62]	54 [49–62]
Cstat H0 post-VHS	108 [106–112]	80 [65–88]	78 [70–83]	101 [97–103]	60 [56–65]	64 [57–68]
Cstat H5	82 [75–82]	49 [41–54]	43 [38–50]	78 [71–92]	41 [38–47]	43 [42–50]
Cdyn H0 pre-VHS	32 [30–35]	22 [18–25]	24 [23–26]	31 [27–35]	23 [20–27]	26 [22–28]
Cdyn H0 post-VHS	53 [49–57]	32 [30–36]	34 [30–36]	53 [49–54]	32 [27–40]	35 [31–37]
Cdyn H5	21 [19–23]	13 [12–14]	13 [11–14]	22 [19–28]	12 [12–14]	15 [13–15]
Edyn H0 pre-VHS	31 [29–33]	45 [40–55]	44 [39–46]	33 [29–37]	43 [37–49]	39 [37–46]
Edyn H0 post-VHS	19 [18–20]	32 [28–33]	30 [29–33]	19 [19–21]	32 [25–37]	29 [27–33]
Edyn H5	48 [45–53]	78 [72–86]	75 [72–90]	47 [36–52]	82 [71–85]	68 [67–75]

Data are presented as median [25th–75th percentile]

Abbreviations: *Peak Pr* peak inspiratory pressures, *Cstat* quasi-static compliance of the respiratory system (calculated using a pressure-volume curve), *Cdyn* dynamic compliance of the respiratory system (calculated using the single frequency forced oscillation technique), *Edyn* dynamic elastance of the respiratory system (calculated using the single frequency forced oscillation technique). Volume history standardization (VHS) consisted in two inflations to an airway pressure of 30 cmH2O. *N* = 8 to 10 animals per group

At initiation of mechanical ventilation, LPS aspiration was associated with an alteration of respiratory mechanics (lower quasi-static and dynamic compliances, higher dynamic elastance and peak inspiratory pressure) as compared to saline aspiration in both WT (WT_(LPS+MV)_ *vs.* WT_(MV)_) and IL-6KO mice (IL-6KO_(LPS+MV)_ *vs.* IL-6KO_(MV)_). These differences were not altered by injections of rHuIL-6. We assessed the relative decrease in the quasi-static compliance during the ventilator protocol as previously described. We found a greater alteration in lung compliance in mice exposed to LPS + MV as compared to mice exposed to mechanical ventilation alone (−25.9% [−31.7;−15.1] in WT_(MV)_ *vs.* −37.4% [−40.0;−35.4] in WT_(LPS+MV)_, *p* = 0,001; (−18.7% [−29.7;−9.8] in IL-6KO_(MV)_ *vs.* (−33.3% [−37.7;−28.1] in IL-6KO_(LPS+MV)_, *p* = 0.011), without any difference between WT and IL-6KO mice.

During mechanical ventilation, dynamic elastance and peak airways pressures increased, whereas dynamic and quasi-static compliances decreased. The progressive deterioration of respiratory mechanics was seen in both IL-6KO and WT mice but was greater in LPS-challenged mice (WT_(LPS+MV)_ and IL-6KO_(LPS+MV)_) as compared to saline-treated mice (WT_(MV)_ and IL-6KO_(MV)_). Furthermore, the progressive deterioration of respiratory mechanics was faster in IL-6KO_(LPS+MV)_ mice as compared to WT_(LPS+MV)_ mice, as shown by differences in dynamic elastance at H1 (65 [59–83] *vs.* 56 [48–60]; *p* = 0.016) (Fig. 2d). These differences were not altered by injections of rHuIL-6.

### Lung inflammation, edema and injury

**Fig. 3:**
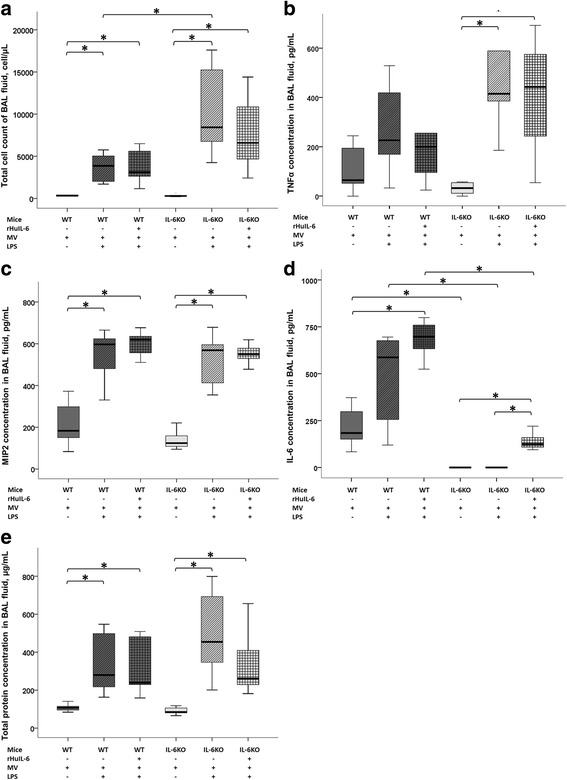
Bronchoalveolar lavage data at the end of a 5-h low tidal volume mechanical ventilation (MV) following either a lipopolysaccharide (LPS) or vehicle (saline) aspiration in wild type (WT) and interleukin-6 knock-out (IL-6KO) mice receiving either recombinant human interleukin-6 (rHuIL-6) or vehicle (saline). **a** Total cell count of BAL fluid (cell/μL). **b** TNFα concentration in the BAL fluid (pg/mL). **c** MIP-2 concentration in the BAL fluid (pg/mL). **d** IL-6 concentration in the BAL fluid (pg/mL). **e** Total protein concentration in the BAL fluid (μg/mL). The symbol * denotes Benjamini-Hochberg corrected *p* value < 0.05 of Mann–Whitney pairwise comparisons (following Kruskal Wallis test). Data are presented as box and whiskers plots. *N* = 6 to 8 animals per group

**Fig. 4:**
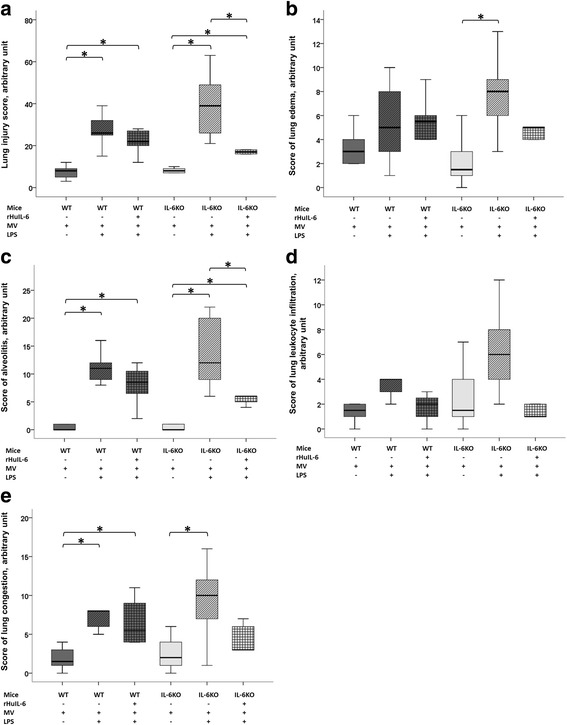
Lung histological analysis at the end of a 5-h low tidal volume mechanical ventilation (MV) following either a lipopolysaccharide (LPS) or vehicle (saline) aspiration in wild type (WT) and interleukin-6 knock-out (IL-6KO) mice receiving either recombinant human interleukin-6 (rHuIL-6) or vehicle (saline). **a** Global lung injury score. **b** Lung edema score. **c** Alveolitis score. **d** Lung leukocyte infiltration score. **e** Lung congestion score. The symbol * denotes Benjamini-Hochberg corrected *p* value < 0.05 of Mann-Whitney pairwise comparisons (following Kruskal Wallis test). Data are presented as box and whiskers plots. *N* = 6 to 8 animals per group

**Fig. 5:**
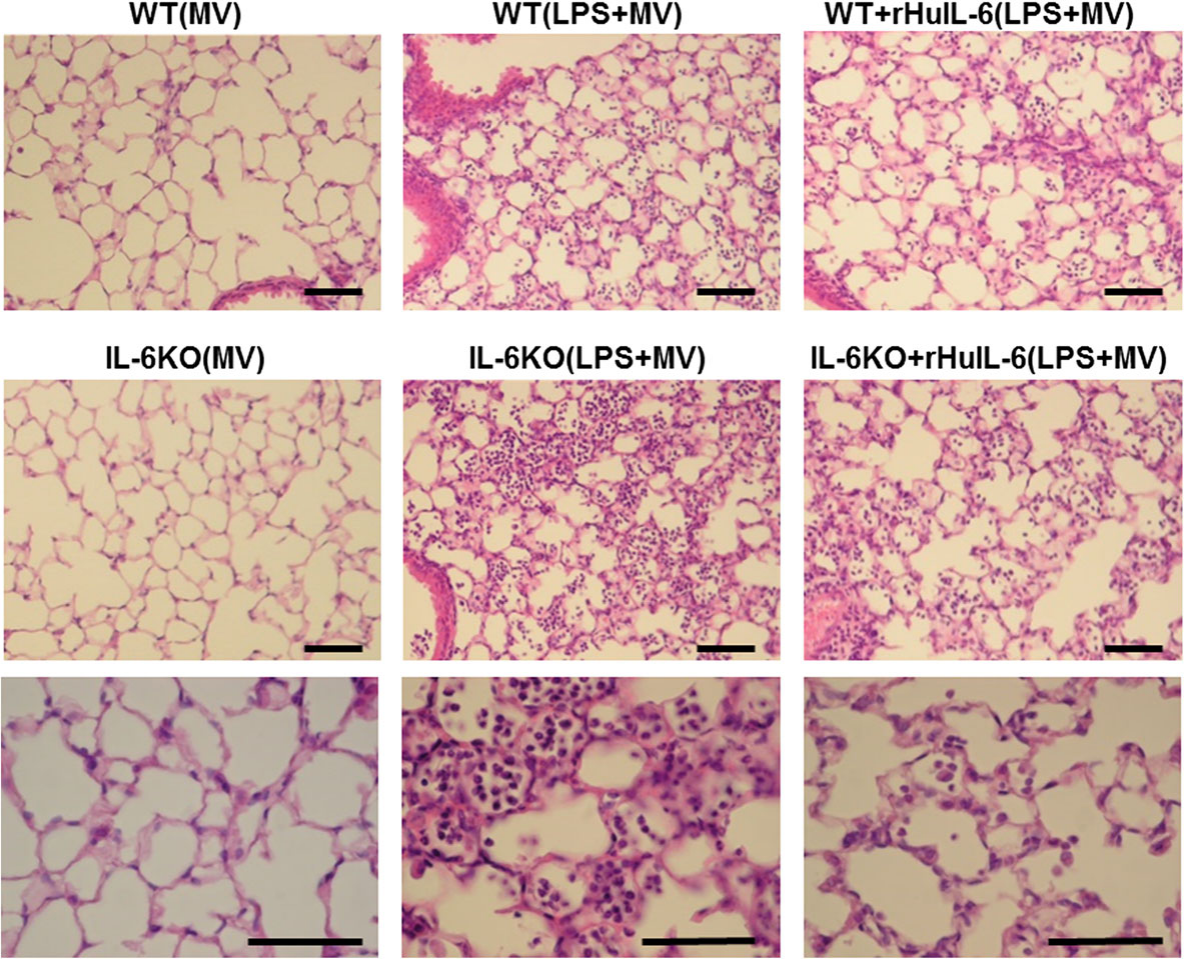
Lung histological sections after a 5-h low tidal volume mechanical ventilation (MV) following either a lipopolysaccharide (LPS) aspiration or vehicle (saline) in wild type (WT) and interleukin-6 knock-out (KO) mice receiving either recombinant human interleukin-6 (rHuIL-6) or vehicle. Lungs underwent fixation (paraformaldehyde 4%), paraffin embedding, staining with hematoxylin and eosin, and examination under a light microscope. Magnification x10 on the two uppers rows of images and X40 on the lower row of pictures. Scale bar: 50μm. *N* = 6 to 8 animals per group

BAL fluid and lung histology data are reported in Figs. 3 and 4, respectively. Lung histology is illustrated in Fig. 5. No differences were observed between IL-6KO_(MV)_ and WT_(MV)_ mice, except for the IL-6 concentration in BAL fluid.

As compared to their saline-treated controls (WT_(MV)_ and IL-6KO_(MV)_), LPS-challenged mice (WT_(LPS+MV)_ and IL-6KO_(LPS+MV)_) showed higher lung inflammation (as assessed by total cell count and MIP2 concentration in BAL fluid, and histological score of alveolitis, Figs. 3a, c and 4C), lung edema (as assessed by total protein concentration in BAL fluid and histological score of lung congestion, Figs. 3e and 4e), and lung injury (as assessed by global lung injury score, Fig. 4a). The differential cell count in WT_(LPS+MV)_ mice and IL-6KO_(LPS+MV)_ mice showed a strong predominance of neutrophils in both groups (95% [94–97,5] and 98% [93–99], respectively; *p* = 0.28). IL-6 was detectable only in all WT groups and in the IL-6KO + rHuIL-6_(LPS+MV)_ group (Fig. 3d).

As compared to WT_(LPS+MV)_ mice, IL-6KO_(LPS+MV)_ mice were more prone to lung inflammation and injury, as shown by a higher total cell count in BAL fluid (8440 [5500–16300)] *vs.* [3860 [1830–5760]; *p* = 0.008) (Fig. 3a) and a trend toward a higher global lung injury score (39 [24–50] *vs.* 26 [24–35]; *p* = 0.190). As compared to IL-6KO_(LPS+MV)_ mice, IL-6KO + rHuIL-6_(LPS+MV)_ mice experienced less severe lung injury, as shown by a lower score of alveolitis [6.0 [4.5–6.5] *vs.* 12.0 [8.0–12.0]; *p* = 0.007) (Fig. 4c), a lower global lung injury score (17 [13–23] *vs.* 39 [24–50]; *p* = 0.007) (Fig. 4a) and a trend toward lower total protein concentration (261 [201–574] *vs.* 455 [311–719]; *p* = 0.181) and lower scores of lung edema (5.0 [3.0–6.5] *vs.* 8.0 [6.0–10.5]; *p* = 0.129), leukocyte infiltration (1.0 [1.0–4.5] *vs.* 6.0 [3.0–10.0]; *p* = 0.081) and congestion (3.0 [3.0–6.5] *vs.* 10.0 [5.0–12.0]; *p* = 0.083).

### Hemodynamics

**Fig. 6:**
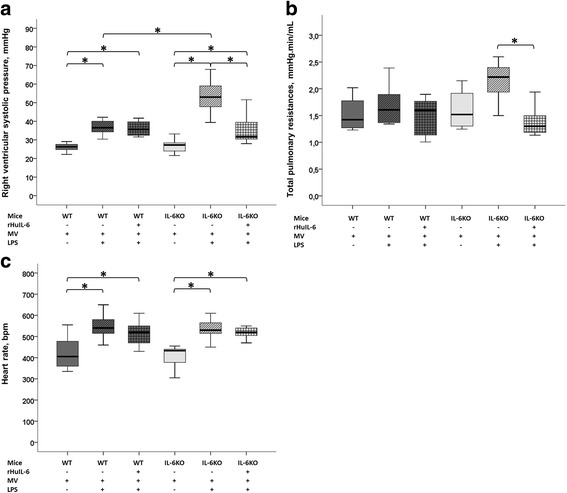
Hemodynamic data obtained at the end of a 5-h low tidal volume mechanical ventilation (MV) following either a lipopolysaccharide (LPS) or vehicle (saline) aspiration in wild type (WT) and interleukin-6 knock-out (IL-6KO) mice receiving either recombinant human interleukin-6 (rHuIL-6) or vehicle (saline). **a** Right ventricular systolic pressure (mmHg). **b** Total pulmonary resistances (mmHg.min/mL). **c** Heart rate (bpm). Right ventricular systolic pressure was measured during a short end-expiratory pause using an ultra-miniature catheter inserted into the right jugular vein and advanced into the right ventricle. Cardiac output was measured by the transpulmonary thermodilution technique. Total pulmonary resistances were calculated as the ratio of right ventricular systolic pressure to cardiac output (measured by the transpulmonary thermodilution technique). The symbol * denotes Benjamini-Hochberg corrected *p* value < 0.05 of Mann–Whitney pairwise comparisons (following Kruskal Wallis test). Data are presented as box and whiskers plots. *N* = 6 to 8 animals per group

Hemodynamics results are shown in Fig. 6. As compared to saline-treated controls, LPS-challenged mice showed higher heart rate and right ventricular systolic pressure (WT_(LPS+MV)_ *vs.* WT_(MV)_ and IL-6KO_(LPS+MV)_ *vs.* IL-6KO_(MV)_). IL-6KO_(LPS+MV)_ mice had a markedly increased right ventricular systolic pressure (53 [47–60] *vs.* 37 [33–41]; *p* < 0.001) (Fig. 6a) and a trend toward a higher total pulmonary resistance (2.21 [1.78–2.41] *vs.* 1.61 [1.37–1.91]; *p* = 0.09) (Fig. 6b) as compared to WT_(LPS+MV)_ mice. These differences were completely abolished in IL-6KO mice treated with rHuIL-6 (IL-6KO + rHuIL-6_(LPS+MV)_ *vs.* WT_(LPS+MV)_).

### Nitric oxide pathway studies

**Fig. 7:**
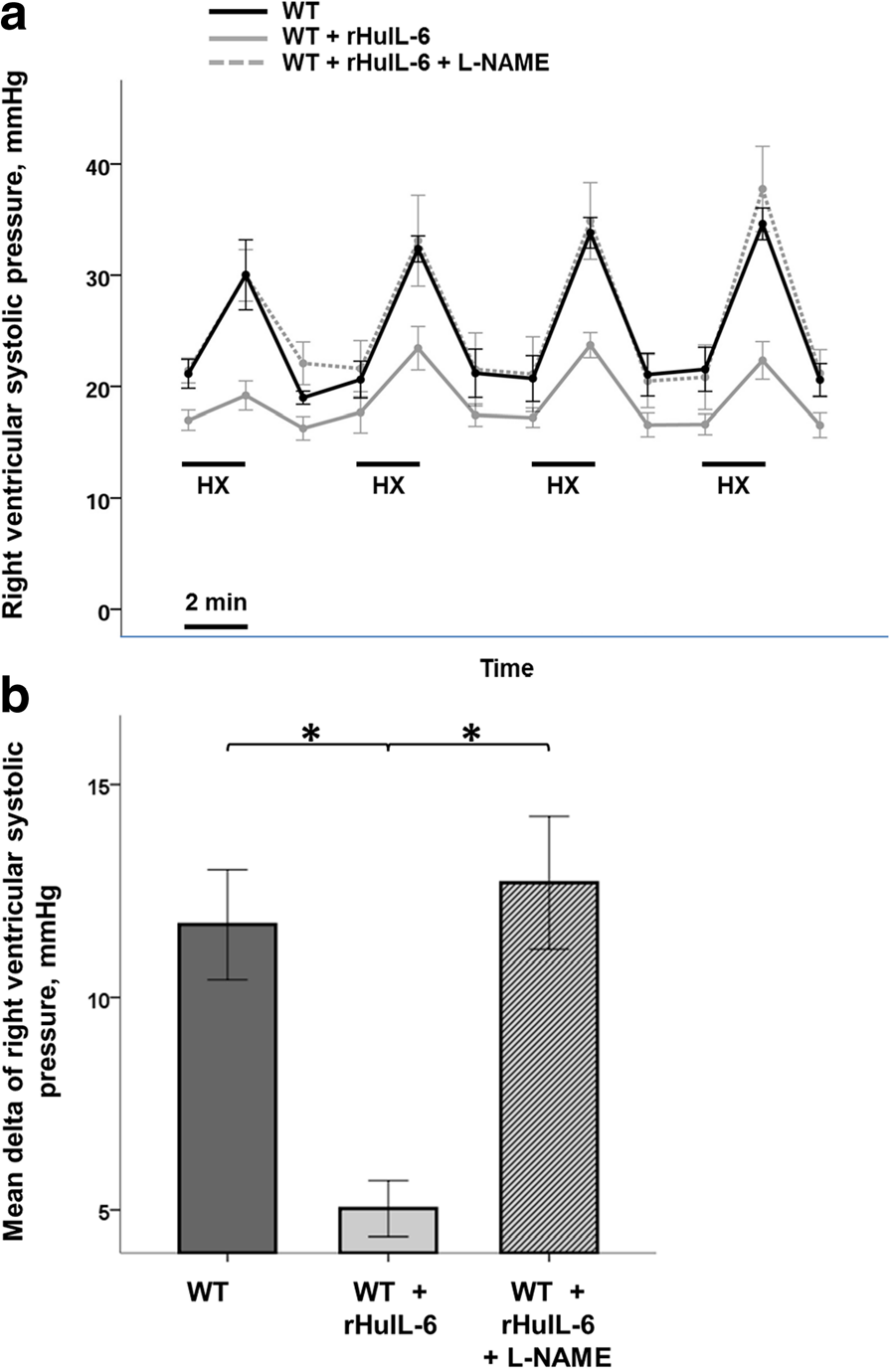
Hemodynamic data obtained during hypoxic challenges. WT mice received either recombinant human interleukin-6 (rHuIL-6) or rHuIL-6 in association with the non-specific inhibitor of nitric oxide synthase Nω-nitro-L-arginine methyl ester hydrochloride (L-NAME). Control mice received vehicle (saline). Five hours later, mice were intubated for mechanical ventilation and an ultra-miniature catheter was immediately inserted into the right jugular vein and advanced into the right ventricle. **a** After 30 min of stabilization, the right ventricular systolic pressure was continuously recorded during four consecutive hypoxic challenges (FiO2 of 0.08 during 2 min, followed by 5 min of reoxygenation). Points represent the mean and errors bars represent the standard error of the mean. **b** The delta of right ventricular systolic pressure was calculated as the mean difference from peak to baseline right ventricular systolic pressure during the four consecutive hypoxic challenges. The symbol * denotes Benjamini-Hochberg corrected *p* value < 0.05 of the Mann–Whitney pairwise comparisons (following Kruskal Wallis test). Columns represent the mean and error bars represent the standard error of the mean. *N* = 4 animals per group

To assess whether the acute pulmonary hypertension observed in IL-6KO mice involved the nitric oxide (NO) pathway, we measured right ventricular systolic pressure (RVSP) during four consecutive periods of hypoxia in WT mice previously treated with rHuIL-6, rHuIL-6 + L-NAME or vehicle. Mice treated with rHuIL-6 showed significantly lower increases in RVSP in response to acute hypoxia as compared to vehicle-treated mice (Fig. 7). The lesser hypoxia-induced pulmonary vasoconstriction was fully prevented by L-NAME administration.

## Discussion

We assessed the right-sided hemodynamic and pulmonary effects of IL-6 deficiency in a septic-like double-hit murine model of ARDS. As compared to WT_(LPS+MV)_ mice, IL-6KO_(LPS+MV)_ mice showed a higher BAL cell count, a faster worsening of lung elastic properties during low tidal volume ventilation, and an acute pulmonary hypertension, the latter at least partially attributable to NO-dependent vaso-active mechanisms. IL-6KO + rHuIL-6_(LPS+MV)_ mice experienced less severe lung injury, as shown by lower alveolitis and global lung injury scores, and a lower total pulmonary resistance as compared to IL-6KO_(LPS+MV)_ mice.

The role of IL-6 in ARDS has been actively investigated over the past decades in various rodent models, including hyperoxia [14, 26], inhalation or aspiration of bacterial particles [15, 27], IgG aspiration [28], acid aspiration [29, 30], bacterial pneumonia [16], LPS intratracheal instillation [31] or aerosol inhalation [15] and high tidal volume-associated lung injury [30, 32]. The role of IL-6 was evaluated using IL-6 overexpression [14, 26], recombinant IL-6 administration [15, 16, 28, 32], IL-6-blocking antibodies [28, 32] and IL-6 deficiency [15, 16, 29–32]. However, none of these studies included a model combining septic-like lung injury and protective mechanical ventilation. In the present work, we used a model clinically relevant to ARDS, the double-hit lung injury model [22], which combines LPS aspiration and low tidal volume ventilation. Lung infection is the most frequent cause of ARDS in humans [1]; low tidal volume ventilation has been shown to be protective during ARDS [33] and is commonly used in clinical practice [34].

Although no formal recommendations are available for ventilatory parameters in mice, we selected a tidal volume of 8 mL/kg, a value that was previously used by several research groups in murine protective mechanical ventilation [35–37]. The duration of low tidal volume mechanical ventilation was adequate as a hit in mice based on previously published work [8]. Our results support the merits of combining LPS and low tidal volume mechanical ventilation to elicit lung inflammation, injury, and an increase in alveolar-capillary permeability in mice. The double hit lung injury model is also characterized by acute pulmonary hypertension [22] and can be used to assess the consequences of IL-6 deficiency on the pulmonary vascular abnormalities typically seen in ARDS. To this end, we elected to use the model in IL-6 deficient mice and their control wild-type littermates. Surprisingly, we found the IL-6 deficiency to be associated with a lower baseline quasi-static compliance in healthy and LPS-exposed animals. To the best of our knowledge, this phenotype of altered elastic lung properties had not been previously reported. Only one study by Tanaka et al. mentions a trend towards a lower lung compliance in IL-6 deficient mice as compared to wild-type controls [38].

Except for three studies [29–31], most investigations have shown that IL-6 exerts lung protective effects in ARDS [14–16, 26–28, 32]. In our study, there was a progressive worsening of elastic lung properties in wild-type and IL6-KO mice during low tidal volume ventilation, probably because of atelectasis [22, 39]. A new finding from our work is a faster worsening of lung elastic properties in IL-6-KO_(LPS+MV)_ mice as compared to WT_(LPS+MV)_. Furthermore, IL-6KO mice experienced more severe pulmonary inflammation and injury in response to LPS aspiration and mechanical ventilation as compared to wild-type animals. The worst scores of edema, congestion, leukocyte infiltration and alveolitis, and the worst global lung injury score were actually seen in IL-6KO_(LPS+MV)_ mice. In agreement with previous reports [32], treatment with exogenous IL-6 reduced histological lung damage in IL-6KO animals and lessened lung leukocyte infiltration in wild-type mice following challenge with LPS and mechanical ventilation. Furthermore, IL-6KO + rHuIL-6_(LPS+MV)_ mice showed a trend toward a lower total protein concentration in BAL fluid and lung edema score when compared to IL-6KO_(LPS+MV)_ mice. This observation is consistent with previous reports showing that IL-6 decreases alveolar permeability and lung edema during lung injury [28, 32]. In contrast, exogenous IL-6 did not reverse the heightened BAL inflammation observed in IL-6KO mice challenged with LPS and mechanical ventilation. Taken together, these data suggest differential effects of IL-6 in lung tissue and alveolar spaces [32].

Protective effects of IL-6 have been mostly reported in septic and septic-like induced ARDS. In addition to promoting inflammation, lung damage and edema, IL-6 deficiency alters bacterial clearance in experimental pneumonia [16, 40] and is associated with increased mortality following LPS aspiration [15]. Furthermore, IL-6 exerts protective effects in extra-pulmonary sepsis models partly through its ability to inhibit apoptosis [20] and promote regeneration of hepatocytes [41]. Intratracheal treatment with recombinant IL-6 has been shown to exert anti-inflammatory effects in mice exposed to intratracheal LPS, with reduced BAL fluid cytokines and lung inflammation [42]. However, results in this research area have not been consistent. Two previous studies in non-septic models of ARDS showed IL-6 had deleterious properties [29, 30]. Another investigation in a double-hit model reported conflicting results. Wild-type mice and IL-6KO mice were subjected to low-dose intratracheal LPS instillation (0,2mg/kg) and 4-h high tidal volume (20mL/kg) mechanical ventilation alone and in combination. As compared to wild-type mice, IL-6KO mice in the combined group showed a significant decrease in bronchoalveolar cellular inflammation and lower histologic scores of lung injury [31]. However, the authors also reported a paradoxical increase in BAL fluid total protein in IL-6KO mice exposed to LPS alone as compared to their WT controls, a finding consistent with our own data. These conflicting observations suggest animal models are important factors to take into consideration when investigating IL-6 effects in ARDS.

ARDS may cause acute pulmonary hypertension [43], a condition associated with poor prognosis in patients [17, 44–46] and potentially worsened by mechanical ventilation, particularly when high PEEP [47] or high-frequency oscillatory ventilation [48] is used. The mechanism of this pulmonary vascular dysfunction is multifactorial, including partially dysregulated vasoreactivity [49]. In IL-6KO_(LPS+MV)_ animals, we observed an increase in total pulmonary resistance, as compared to WT_(LPS+MV)_ mice and IL-6KO + rHuIL-6_(LPS+MV)_ mice. We hypothesized that the acute pulmonary hypertension observed in our study could be related to a dysregulation of NO-dependent vasoactive mechanisms. Several studies have shown that IL-6 exerts acute systemic hemodynamics effects through this pathway. The acute administration of IL-6 produced a transient dose-dependent systemic hypotension in rats [50]. In vitro, acute exposure of hamster papillary muscles [51] or cardiomyocytes [52] to IL-6 reduced contractility. This acute negative inotropic effect may result from an activation of endothelial NO synthase (eNOS) and inducible NO synthase (iNOS) [53], with a downstream decrease in intracellular Ca^++^ influx [54]. Other studies have suggested that IL-6, in association with IL-1β, is implicated in the NOS-dependent systemic vasodilatation seen during sepsis [55]. In the present study, rHuIL-6 treatment in healthy wild-type, hypoxia-challenged mice reduced hypoxic pulmonary vasoconstriction. This result is in agreement with in another paper by our team, which described a trend toward a higher hemodynamic response to acute hypoxia (5 min) in IL-6KO mice as compared to WT mice; conversely, IL-6-KO mice exhibited a lower right ventricular systolic pressure in response to chronic hypoxia (2 weeks) as compared to WT mice [56].

Because both eNOS and iNOS contribute to pulmonary vascular tone and modulate hypoxic pulmonary vasoconstriction [57], we tested the effects of the non-selective NOS inhibitor L-NAME. Its co-administration with rHuIL-6 totally restored hypoxic pulmonary vasoconstriction. This finding suggests a role for NOS isoforms in mediating the IL-6-induced inhibition of hypoxic pulmonary vasoconstriction. Therefore, the acute pulmonary hypertension observed during lung injury in LPS-exposed mice lacking endogenous IL-6 may be attributed in part to vasoactive mechanisms involving a dysregulation of NOS isoforms. This hypothesis will have to be fully investigated in future studies. Of note, the effects of IL-6-related inhibition of hypoxic pulmonary vasoconstriction may be considered either beneficial on right-sided hemodynamics or deleterious on gas exchange. The clinical relevance of these changes must be kept in mind when debating the potential therapeutic applications of rHuIL-6 in patients with ARDS.

The hemodynamic changes observed in IL-6KO_(LPS+MV)_ mice may cause further lung injury and/or inflammation via a direct mechanism. Several studies support this conclusion. Following pulmonary artery banding to induce pre-capillary pulmonary hypertension and subsequent right heart failure in mice, Vistnes et al. quantified circulating cytokines levels and observed an increase in IL-1α, IL-6, G-CSF and CXCL9 as compared to sham operated mice [58]. In another study, Costa-Souza et al. induced pre-capillary pulmonary hypertension and quantified pulmonary inflammation in a rat model of microsphere-induced pulmonary embolism. The authors observed an increase in bronchoalveolar neutrophils as compared to control rats [59]. Taken together, the results from these two non-septic rodent models of acute pre-capillary pulmonary hypertension suggest the elevated right ventricular systolic pressure (or pulmonary arterial pressure) might contribute directly to pulmonary and systemic inflammation. Previous work from our research group suggests additional factors may be involved. In a LPS-challenged mice model, 5-h mechanical ventilation using low tidal volume inflation alone or in combination with intermittent deep inflations produced a significant rise in right ventricular systolic pressures and pulmonary vascular resistance. However, we did not observe any increase in lung and systemic inflammation [22].

Our study has several limitations. First, we did not measure systemic blood pressure, and assumed no differences were present between groups. This assumption is reasonable considering previously available results showing that baseline systemic blood pressure is similar in WT and IL-6KO mice [60]. Second, we also assumed that left-sided myocardial function and pressures did not differ between groups. This assumption is supported by previous findings that intravenous exogenous IL-6 exerts only very transient left-sided hemodynamic effects in rodents [50].

## Conclusions

In a septic-like double-hit murine model of ARDS (LPS aspiration followed by 5-h low tidal volume mechanical ventilation), we found that IL-6 deficiency caused a more severe bronchoalveolar cellular inflammation and a faster deterioration of lung elastic properties during low tidal volume ventilation. This higher severity was partially reversed by exogenous IL-6, which mitigated the histological injury observed in IL-6 deficient mice challenged with LPS and mechanical ventilation. Furthermore, IL-6 deficiency was associated with marked acute pulmonary hypertension, which was prevented by exogenous IL-6 administration. IL-6 acted as limiting the hypoxic pulmonary vasoconstriction, most probably by regulating the NO pathway. Future studies are needed to reconcile the discrepancy between the commonly recognized merits of using IL-6 concentrations as a detrimental prognostic factor in sepsis in a clinical setting and its protective role in experimental sepsis and ARDS.

## Abbreviations

ALI: Acute lung injury
ARDS: Acute respiratory distress syndrome
BAL: Broncho-alveolar lavage
eNOS: Endothelial nitric oxyde synthase
IL-6: Interleukin-6
IL-6KO: Interleukin-6 knock-out
iNOS: Inducible nitric oxyde synthase
L-NAME: Nω-nitro-L-arginine methyl ester hydrochloride
rHuIL-6: Recombinant human interleukin-6
RVSP: Right ventricular systolic pressure
WT: Wild-type
